# The Relationship Between Parental Satisfaction and Parental Loyalty in Kindergartens: The Mediating Role of Parental Trust and Parental Relationship Commitment

**DOI:** 10.3389/fpsyg.2022.822164

**Published:** 2022-04-07

**Authors:** Jian-Hao Huang, Ling-Ge Chen, Yan-Xia Ma, Sun-Yu Gao

**Affiliations:** Dhurakij Pundit University, Bangkok, Thailand

**Keywords:** parental satisfaction, parental loyalty, parental trust, parental relationship commitment, KMV model

## Abstract

This study aimed to explore the psychological mechanisms behind the relationship between kindergarten parental satisfaction and parental loyalty. This study used the parental satisfaction scale, parental trust scale, parental relationship commitment scale, and parental loyalty scale on 923 kindergarten parents. The test was conducted on 923 kindergarten parents. The results of this study showed that parental satisfaction significantly and positively affected parental loyalty. Parental trust was partially mediated between parental satisfaction and parental loyalty. Parental relationship commitment was also partially mediated between parental satisfaction and parental loyalty. Regarding to parental satisfaction and parental loyalty, parental trust and parental relationship commitment had chain mediation effect between parental satisfaction and parental loyalty. The findings of this study provided valuable insights into the effect of parental satisfaction on parental loyalty and offer concrete practical suggestions for kindergarten operators to improve the loyalty of kindergarten parents.

## Introduction

In business management, attracting new customers and maintaining existing customers are two issues that have always existed. In the past, companies have focused on developing new customers at the expense of maintaining existing ones. Past research has shown that it cost five times more to develop a new customer than it did to retain an existing one (Hart et al., 1990). Furthermore, if a company could increase its customer retention rate by 5%, it could increase its profitability by 25–85% (Reichheld and Sasser, 1990). Thus, an increase in customer loyalty can lead to significant cost savings and greater profitability for the business. As parents are the main customers of kindergartens, building and maintaining good relationships with parents and increasing parental loyalty are important issues for the sustainability of kindergartens in an increasingly competitive early childhood education market.

Among the various factors associated with customer loyalty, customer satisfaction has been shown to be an important predictor of customer loyalty. For example, Ling and Ding (2006) found that increasing customer satisfaction was effective in promoting customer loyalty, which gave firms an advantage in survival and competition. Han et al. (2021) also found that satisfaction had a positive and significant effect on loyalty. Similar findings are now available in the field of education. In a study of university students, Chen (2013) found that when students’ satisfaction with their school increased, so did their loyalty to the school. Río-Rama et al. (2021) pointed out parental satisfaction had a significant and positive effect on parental loyalty. Budiyanto et al. (2021) showed that the higher the kindergarten parental satisfaction, the higher the parental loyalty. Research has established that satisfaction affected loyalty. However, the characteristics of educational organizations are different from those of business organizations. Despite the not-for-profit nature of educational organizations, few studies have examined the effect of parental satisfaction on parental loyalty. In kindergartens, the operators and teachers of the kindergartens have to meet the needs of the children as well as the satisfaction and loyalty of the parents. Therefore, this study will examine the relationship between parental satisfaction and parental loyalty using kindergarten parents as the study object, in the hope of obtaining more empirical evidence.

According to the Key Mediating Variable model (KMV model), trust and relationship commitment were key mediating factors for relationship marketing success (Morgan and Hunt, 1994). We also note that current research reported that satisfaction had a significant and positive effect on trust (Song et al., 2019). Trust had a significant and positive effect on loyalty (Jamshidi and Rousta, 2021). There was a significant and positive effect of satisfaction on relationship commitment (Nascimento and Little, 2020). There was a significant and positive effect of relationship commitment on loyalty (Zhang et al., 2020). Trust had a significant and positive effect on relationship commitment (Wang and Lin, 2021). In conclusion, existing research supported that satisfaction had a significant and positive effect on loyalty and that trust and relationship commitment had a mediation effect between satisfaction and loyalty (Sanchez-Franco et al., 2009). However, the internal mechanisms of trust and relationship commitment between parental satisfaction and parental loyalty in educational organizations are not yet clear. In particular, kindergartens are important places for children to have fun and learn. Children spend a lot of time in kindergartens, and if the services and education provide by kindergartens gain the trust and relationship commitment of parents, then the loyalty of kindergarten parents may increase as a result of increased satisfaction. This is an important task for kindergarten operators. From this perspective, it is hypothesized that parental trust and parental relationship commitment may be important mediating variables between kindergarten parental satisfaction and parental loyalty.

Therefore, this study aims to examine the impact of parental satisfaction on parental loyalty from the perspective of kindergarten parents, and the mediating role of parental trust and parental relationship commitment between parental satisfaction and parental loyalty. This results will enable us to better understand the important factors that influence parental loyalty in kindergartens, which will increase our awareness of the potential impact mechanisms of the process and provide new directions on how kindergarten operators can more effectively promote parental loyalty.

## Literature Review

### Satisfaction and Loyalty

Satisfaction is an experience that consumers had with a product or service, and the smaller the gap between consumers’ expectations and their actual feelings, the higher the satisfaction (Kotler and Armstrong, 1996). When this concept was applied to the education industry, satisfaction was an outcome of the evaluation of educational services and a short-term attitude (Elliott and Healy, 2001). This study defines kindergarten parental satisfaction as parents’ actual feelings toward kindergarten education services. Sirdeshmukh et al. (2002) proposed that loyalty was a behavior that indicates an expectation to maintain a buying relationship with the selling company, including a high purchase rate for a particular provider, positive word-of-mouth communication, and repeat purchases. Hsieh and Lin (2011) defined parental loyalty was parent’s desire to let their children study in the school they expected. On the other hand, they recommend the school to other parents because of the circumstances of their children’s attendance and their interactions in school. Children would be sent to the same school even when there was a change in staffing or external environment. Leventhal et al. (2006) conducted a survey of 1,003 parents in childcare settings, the results showed that parental satisfaction with the childcare center positively influenced loyalty. This means that the more satisfied parents are, the more likely they are to keep their children in school. Previous empirical studies have found that satisfaction has a significant and positive effect on loyalty (Cabello-Manrique et al., 2021; Han et al., 2021; Río-Rama et al., 2021). Budiyanto et al. (2021) showed that parental satisfaction had a significant and positive effect on parental loyalty. It is found that the higher the parental satisfaction in kindergarten, the higher the parental loyalty. Therefore, this study proposes the following H1: Parental satisfaction in kindergarten has a significant and positive effect on parental loyalty.

### Satisfaction, Trust, and Loyalty

Morgan and Hunt (1994) identified trust as the degree to which relationship members had confidence in the reliability and honesty of their trading partners. According to Dwyer and Lagace (1986), trust was an expectation of faith, confidence, and trading partners, and a basis for future behavior. In this study, parental trust is defined as parents’ confidence and expectations of kindergartens. Garbarino and Johnson (1999) suggested that satisfaction was a prerequisite for trust. In a study of Chinese consumers, Song et al. (2019) found that satisfaction was one of the most important factors in enhancing trust. The higher the satisfaction, the higher the trust in the brand. Høgevold et al. (2020) similarly mentioned in their study of Norwegian salespeople that the higher the salesperson’s satisfaction with the customer, the higher the trust in the customer. In the education literature, research by Schlesinger et al. (2017) demonstrated that graduates’ satisfaction with their school could positively influence their loyalty to the university. Kindergarten parental satisfaction may therefore have a positive effect on parental trust. That is, the higher the satisfaction of the kindergarten parents, the higher the trust in the kindergarten parents is likely to be.

Aydin et al. (2005) mentioned that trust was one of the most important determinants of consumer brand loyalty. Brand loyalty was also a result of trust, which was a key element of brand loyalty (Alhaddad, 2015). Han et al. (2021) found that trust had a positive and significant effect on loyalty. In a study of 520 Malaysian consumers, Jamshidi and Rousta (2021) found that the higher the trust in the brand, the higher the likelihood of repurchase, the higher loyalty they had. In the education literature, Carvalho and de Oliveira Mota (2010) found from a study of 431 college students’ trust in higher education significantly and positively influenced college students’ loyalty. It follows that the higher the trust of parents in kindergartens, the higher the parental loyalty is likely to be.

In summary, satisfaction has the potential to increase trust, which in turn increases loyalty through trust. Therefore, this study proposes H2, which states that as: Parental trust has a mediating role in the effect of kindergarten parental satisfaction on parental loyalty.

### Satisfaction, Relationship Commitment, and Loyalty

Morgan and Hunt (1994) defined relationship commitment as the desire to maintain a relationship of purchase value over time; in other words, a relationship that both parties believe was worth maintaining. Pritchard et al. (1999) proposed that when a company had relationship commitment with its customers, both parties were more willing to establish a long-term stable relationship. Therefore, in this study, parental relationship commitment means that parents are willing to maintain a long-term relationship with the kindergarten. Talukder (2019) found in his study that employees with higher levels of job satisfaction had higher levels of relationship commitment to the organization. In a study of 150 couples, Nascimento and Little (2020) also found that the level of relationship commitment of couples increased with satisfaction. In educational field, Pham and Lai’s (2016) found that university students’ satisfaction with their school significantly and positively influenced commitment. Therefore, an increase in the satisfaction of kindergarten parents may further enhance parental relationship commitment.

In addition, Rauyruen and Miller (2007) found that relationship commitment had a significant and positive effect on customer loyalty, implying that customers were willing to continue to work with the company and build long-term relationships, and that customer loyalty increased as a result. Zhang et al. (2020) also recommended that the better the consumer perceives of the brand they had, the higher the consumer’s loyalty to the brand they had. In the field of education, Skallerud’s (2011) also confirmed that strengthening parent-school commitment was one of the necessary conditions for solidifying parental loyalty. It can be seen that when parents have a higher relationship commitment to the kindergarten, they are likely to have a higher level of loyalty.

In conclusion, the higher the satisfaction, the higher the relationship commitment, and the higher the relationship commitment, the more positive the effect on loyalty. This study concludes that kindergarten parental satisfaction may increase relationship commitment, which in turn may positively affect kindergarten parental loyalty. Therefore, there may be a mediating role of relationship commitment in kindergarten parental satisfaction and loyalty. H3: Relationship commitment plays a mediating role in the effect of parental satisfaction on loyalty in kindergarten.

### Satisfaction, Trust, Relationship Commitment, and Loyalty

Morgan and Hunt (1994) proposed the KMV model and showed that trust and relationship commitment were the key mediating factors for successful relationship marketing, a partnership characterized by trust, and relationship commitment made both parties more accepting of high-risk situations, as both parties trust each other to engage in activities that maximize long-term benefits. Friman et al. (2002) showed that the KMV model had also been validated in the context of business-to-business relationships, trust and relationship commitment were important mediating variables in the development of relationship marketing between firms. It could be effective in fostering a relationship between two parties and could be beneficial in sustaining long-term cooperation. Sue and Campbell’s (2012) also suggested that the KMV model could also be applied to librarians and teachers. Trust and relationship commitment did facilitate collaborative relationships between librarians and teachers in teaching and research. Wang and Lin (2021) showed that the KMV model could also be applied to students in nursing and medical schools. Interpersonal trust and relationship commitment act as chain mediating roles between interpersonal relationship quality and knowledge sharing behavior. As a result, the KMV model has been applied to a variety of fields and groups, and trust and relationship commitment have generally been shown to play an important role as mediating variables in empirical studies of relationship marketing. Sanchez-Franco et al. (2009) further found in their study that the KMV model could also be applied to customers of Internet services, trust and relationship commitment had mediation effects between satisfaction and loyalty. To summarize, the KMV model may also be applicable to kindergarten parents. Specifically, kindergarten parental satisfaction may have an enhancing effect on parental trust and parental relationship commitment. Parental trust may also increase parental relationship commitment, which in turn may increase parental loyalty. Therefore, this study proposed H4, which stated that kindergarten parental trust and parental relationship commitment play significant chain mediating roles in the relationship between parental satisfaction and parental loyalty.

## Research Methods

### Data Collection and Analysis

First, this study used the Parent Satisfaction Scale, Parent Trust Scale, Parent Relationship Commitment Scale, and Parent Loyalty Scale to conduct a questionnaire survey on parents of a kindergarten in Taiwan. Convenience sampling was used to select 194 parents, including 99 males and 95 females. Exploratory factor analysis and reliability analysis were completed. Then, aiming at other 15 public kindergartens and 16 private kindergartens in Taiwan, we got 923 valid samples. A 462 were males and 461 were females, the statistic was analyzed by confirmatory factor analysis, common method variance test, descriptive statistics, correlation analysis, and testing of chain mediation effect.

### Research Tools

#### Parental Satisfaction Scale

By reference to various measures of parental satisfaction developed by Crosby et al. (1990), Lagace et al. (1991), and McAlexander et al. (2003), this study developed a parental satisfaction scale, which was a 5-point scale, where 1 meant strongly disagree and five meant strongly agree (five questions in total). The higher the score, the higher the parental trust in the kindergarten.

#### Parental Trust Scale

By reference to the literature and measurements of parental trust developed by Crosby et al. (1990), Lagace et al. (1991), and McAlexander et al. (2003), this study developed a parental trust scale, which used a 5-point scale, where one meant strongly disagree and five meant strongly agree (five questions in total). The higher the score, the higher the parental trust in the kindergarten.

#### Parental Relationship Commitment Scale

Taking reference from Crosby et al. (1990), Morgan and Hunt (1994), and McAlexander et al. (2003) on the measurement of parental relationship commitment, this study developed a parental relationship commitment scale used a 5-point scale, one meant strongly disagree, five meant strongly agree (five questions in total). The higher the score, the stronger the relationship commitment between the parents and the kindergarten.

#### Parental Loyalty Scale

With reference to the measurement of loyalty by Zeithaml et al. (1996), Gronholdt et al. (2000), and McAlexander et al. (2003), a parental loyalty scale was developed used a 5-point scale ranging from one strongly disagree to five strongly agree. There were five questions in total, where higher scores indicate higher parental loyalty to the kindergarten.

### Exploratory Factor Analysis

As can be seen from Table 1, this study used pilot test samples for Exploratory Factor Analysis. The results showed that the Kaiser-Meyer-Olkin (KMO) Test = 0.955 and the Bartlett’s Test of Sphericity value were significant at *p* < 0.001. Kaiser and Rice (1974) suggested that when the KMO > 0.8, the significance (*p* < 0.05) data of the value of Bartlett’s Test of Sphericity were suitable for factor analysis. Therefore, this study used the maximum variance rotation method for factor analysis, rotating the component matrices to show that four factors with eigenvalues greater than one were generated. The factor loadings for the four factors ranged from 0.435 to 0.831, which met the criterion that the factor loadings need to be greater than 0.3 (Zaltman and Burger, 1975). The explanation rate of parental satisfaction was 24.25%, parental trust was 20.37%, parental relationship commitment was 19.67%, and parental loyalty was 14.71%.

**Table 1:** Summary table of exploratory factor analysis.

S. No.	Items	Factor loadings			
		Parental satisfaction	Parental trust	Parental relationship commitment	Parental loyalty
Parental satisfaction (explanation rate = 24.25%)					
1.	This kindergarten makes me feel satisfied	0.729			
2.	I feel that the actual cost I pay is worth it	0.760			
3.	My interactions with this kindergarten have always been pleasant	0.795			
4.	The overall performance of this kindergarten is close to my expectations	0.757			
5.	I am glad I chose the right kindergarten	0.722			
Parental trust (explanation rate = 20.37%)					
6.	I believe that the kindergarten is able to handle unexpected situations such as high fever and injuries		0.621		
7.	I believe that the kindergarten cares about parents’ needs for their children’s learning		0.712		
8.	I believe that the kindergarten’s first priority is the children		0.737		
9.	I have confidence in the teaching environment provided by the kindergarten		0.636		
10.	Overall, I think the kindergarten is trustworthy		0.685		
Parental relationship commitment (explanation rate = 19.67%)					
11.	I think it is important to maintain a long-term partnership with the kindergarten			0.435	
12.	I am committed to participating in the activities organized by the kindergarten			0.796	
13.	I am willing to give extra time and effort to the kindergarten			0.795	
14.	I am proud of having my child in this kindergarten			0.454	
15.	I enjoy making friends with the teachers at the kindergarten			0.444	
Parental loyalty (explanation rate = 14.71%)					
16.	I would never consider transferring my child to another kindergarten				0.779
17.	I would recommend this kindergarten to anyone who asks me about it				0.655
18.	I would continue to choose this kindergarten even if the fees of another school were reduced				0.831
19.	I will continue to choose this kindergarten if I have other children who need to go to school				0.666
20.	I would still choose this kindergarten even if the school fees were increased				0.756
**Cumulative explained variance in total = 75.20%**					

### Reliability Analysis

In this study, pilot test samples were used for reliability analysis. The results were as follows: Cronbach’s *α* = 0.944 for the parental satisfaction scale, Cronbach’s *α* = 0.945 for the parental trust scale, Cronbach’s *α* = 0.891 for the parental relationship commitment scale, and Cronbach’s *α* = 0.914 for the parental loyalty scale, indicating that the reliability of the scales was good. In summary, this questionnaire consists of four scales with a total of 20 questions. The total Cronbach’s *α* = 0.967, indicating good reliability of this questionnaire.

## Results

### Measurement Model

Confirmatory Factor Analysis (CFA) was conducted using a formal sample to test the suitability of a measurement model consisting of four interrelated variables: parental satisfaction, parental trust, parental relationship commitment, and parental loyalty. The results of the measurement model data were as follows: *χ*^2^ = 897.959, df = 1,644, *χ*^2^/df = 5.475, CFI = 0.916, TLI = 0.954, SRMR = 0.030, RMSEA = 0.067, indicating a good fit of the measurement model to the observed data. Convergent validity including Average Variance Extracted (AVE) and Construct Reliability (CR). Convergent validity reliabilities for each variable of the scale were as follows: AVE of parental satisfaction = 0.753, CR of parental satisfaction = 0.938; AVE of parental trust = 0.758, CR of parental trust = 0.940; AVE of parental relationship commitment = 0.604, CR of parental relationship commitment = 0.883; and AVE of parental loyalty = 0.700, CR of parental loyalty = 0.921, indicating good convergent validity for all variables.

### Common Method Variance

In this study, Common Method Variance (CMV) was conducted by comparing the fitness of the multi-factor CFA with the single-factor CFA (Podsakoff and Organ, 1986). Table 2 shows that the multi-factor model fits well and has a much lower *χ*^2^ than the single-factor model, which means that the two models are significantly different (*p* < 0.001) and therefore, it is inferred that the common method variation problem is not serious in this study.

**Table 2:** Comparison of single- and multi-factor models.

Pattern	*χ* ^2^	df	Δ*χ*^2^	Δdf	*p*
Single-factor model	2952.185	170	2059.226	6	0.000
multi-factor model	897.959	164			

#### Descriptive Statistics and Correlation Analysis

Pearson’s correlation analysis was conducted used the formal sample and correlations between the variables were observed. Table 3 shows that parental satisfaction and parental trust had a significant and positive correlation (*r* = 0.833, *p* < 0.001). Parental satisfaction and parental relationship commitment had a significant and positive correlation (*r* = 0.751, *p* < 0.001) and parental satisfaction and parental loyalty had a significant and positive correlation (*r* = 0.738, *p* < 0.001). Parental trust and parental relationship commitment had a significant and positive correlation (*r* = 0.767, *p* < 0.001), and parental trust and parental loyalty had a significant and positive correlation (*r* = 0.731, *p* < 0.001). Parental relationship commitment and parental loyalty had a significant and positive correlation (*r* = 0.690, *p* < 0.001). The above correlation coefficients may be highly correlated. Therefore, further tests of discriminant validity can be conducted. The results of the analysis showed that the root AVE values of two variables was greater than the correlation coefficient between the two variables, which met Fornell and Larcker’s (1981) criteria for assessing discriminant validity and indicated good discriminant validity between any two variables.

**Table 3:** Summary table of descriptive statistics and correlation analysis.

Variables	*M*	*SD*	SAT	TRU	R-COM	LOY
SAT	4.32	0.579	**0.868**			
TRU	4.39	0.547	0.833^***^	**0.870**		
R-COM	4.29	0.554	0.751^***^	0.767^***^	**0.777**	
LOY	4.21	0.634	0.738^***^	0.731^***^	0.690^***^	**0.836**

*SAT, parental satisfaction; *TRU*, parental trust; *R-COM*, parental relationship commitment; LOY, parental loyalty; bolded fonts are root AVE values*.

*** *p < 0.001*.

#### Analysis of the Chain Mediation Effect

The PROCESS macro of SPSS was suitable for the analysis of the effects of mediation and also for chain mediation models with multiple mediating variables (Demming et al., 2017). Therefore, the study used Model 6 in PROCESS macro of SPSS (Hayes, 2012) to analyze the chain mediation effect of parental trust and parental relationship commitment between kindergarten parent satisfaction and parental loyalty. Table 4 shows the results of the regression analysis. The results showed that parental satisfaction had a significant and positive predictive effect on parental loyalty in Model 1 (*B* = 0.809, *p* < 0.001) and therefore, H1 is supported. In Model 2, parental satisfaction significantly and positively predicted parental trust (*B* = 0.788, *p* < 0.001). In Model 3, parental satisfaction significantly and positively predicted parental relationship commitment (*B* = 0.350, *p* < 0.001), parental trust significantly and positively predicted parental relationship commitment (*B* = 0.468, *p* < 0.001). In Model 4, parental trust significantly and positively predicted parental loyalty (*B* = 0.321, *p* < 0.001). In addition, parental relationship commitment significantly and positively predicted parental loyalty (*B* = 0.253, *p* < 0.001). In summary, parental trust and parental relationship commitment show significant chain mediation in the effect of kindergarten parental satisfaction on parental loyalty. H2–H4 of this study are therefore supported.

**Table 4:** Model results.

Variables	Model 1		Model 2		Model 3		Model 4	
	LOY		TRU		R-COM		LOY	
	*B*	*t*	*B*	*t*	*B*	*t*	*B*	*t*
Constant	0.711	6.942^***^	0.985	13.603^***^	0.726	8.262^***^	0.094	0.876
SAT	0.809	34.438^***^	0.788	47.434^***^	0.350	10.454^***^	0.374	8.979^***^
TRU					0.468	13.235^***^	0.321	7.092^***^
COM							0.253	6.736^***^
*R^2^*	0.545		0.694		0.630		0.607	
*F*	1185.952^***^		2249.946^***^		842.148^***^		508.898^***^	

*SAT, parental satisfaction; *TRU*, parental trust; *R-COM*, parental relationship commitment; LOY, parental loyalty*.

*** *p < 0.001*.

**Figure 1:**
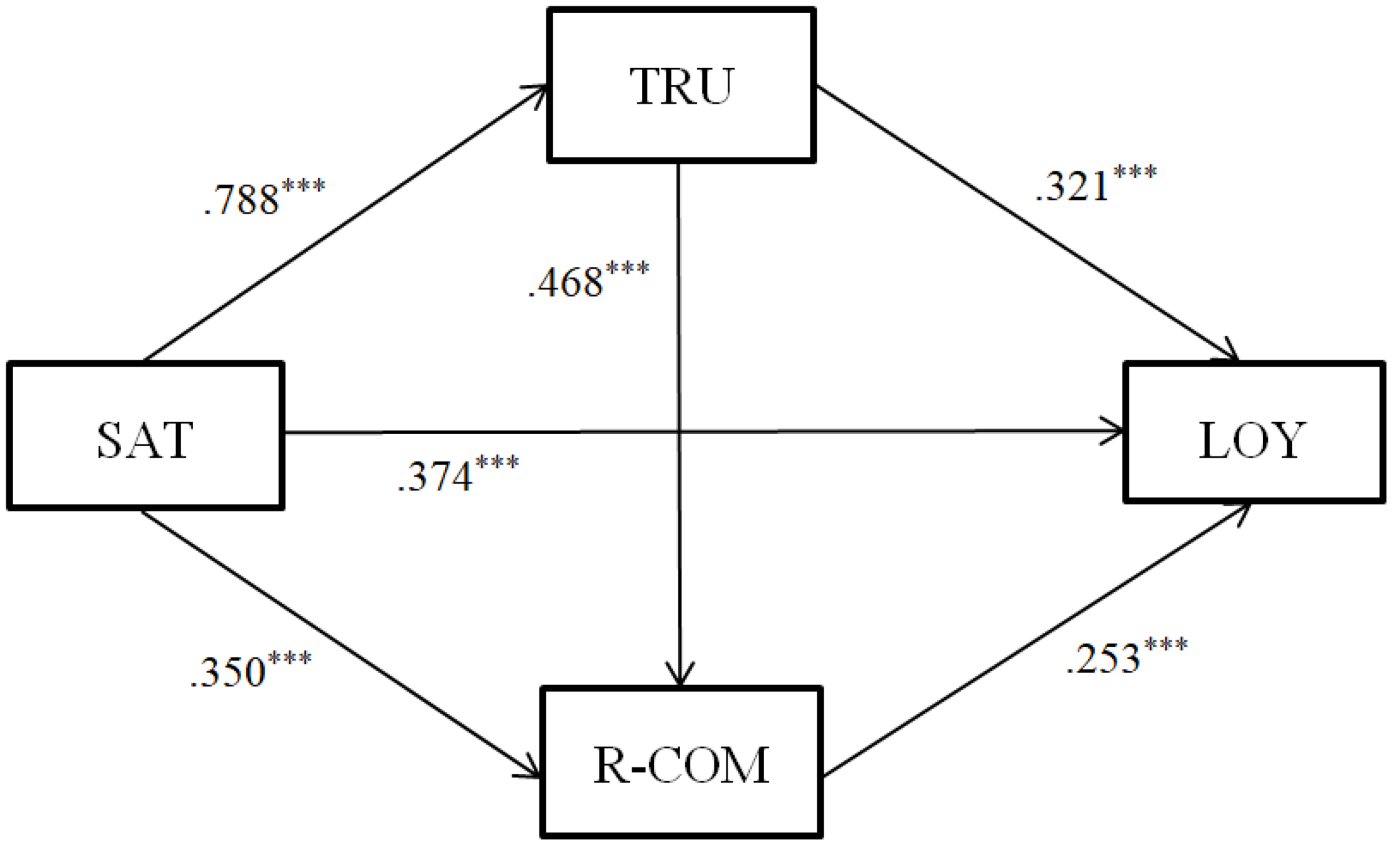
The chain mediation model. SAT, parental satisfaction; TRU, parental trust; R-COM, parental relationship commitment; LOY, parental loyalty; and ^***^p < 0.001.

As suggested by Preacher and Hayes (2008), the stability of the mediation model could be tested using the more robust Bootstrapping method. Table 5 shows the results of the analysis of the chain mediation model used the Bootstrapping method. Figure 1 shows the path diagram and effect values for the chain mediation model. As seen in Table 5, the total indirect effect value was 0.435 and the total indirect effect consists of three indirect effects: Indirect effect path 1: parental satisfaction→parental trust→parental loyalty (indirect effect = 0.253, LLCI = 0.171, ULCI = 0.343). Indirect effect path 2: parental satisfaction→parental relationship commitment→parental loyalty (indirect effect = 0.088, LLCI = 0.048, ULCI = 0.135). Indirect effect path 3: parental satisfaction→parental trust→parental relationship commitment→parental loyalty (indirect effect = 0.093, LLCI = 0.057, ULCI = 0.130). The 95% confidence interval for these indirect effects did not contain 0, indicating that all three indirect effects reached statistical significance. In addition, parental satisfaction had a significant and positive predictive effect on parental loyalty (direct effect = 0.374, LLCI  = 0.292, ULCI = 0.455). Therefore, this suggests that parental trust plays a partial mediation effect in the effect of parental satisfaction on parental loyalty. Parental relationship commitment plays a partial mediation effect in the effect of parental satisfaction on parental loyalty. Parental trust and parental relationship commitment play the chain mediation effect in the effect of parental satisfaction on parental loyalty. Thus, H2–H4 of this study are confirmed (see Figure 1).

**Table 5:** Mediation effect with bootstrapping.

	Effect	SE	95% LLCI	95% ULCI
Direct effect	0.374	0.042	0.292	0.455
Total Indirect effect	0.435	0.043	0.353	0.518
Indirect effect 1	0.253	0.044	0.171	0.343
Indirect effect 2	0.088	0.022	0.048	0.135
Indirect effect 3	0.093	0.019	0.057	0.130

*Bootstrapping random sampling 5,000 times; SE, standard error; LLCI, lower limit of confidence interval; ULCI, upper limit of confidence interval; indirect effect 1 = SAT→ *TRU*→ LOY; indirect effect 2 = SAT→ *R-COM*→ LOY; and indirect effect 3 = SAT→ *TRU*→ *R-COM*→ LOY*.

## Discussion

According to H1, the results of this study suggested that kindergarten parental satisfaction had a significant and positive effect on parental loyalty. Similar to previous research findings, increased satisfaction was associated with increased loyalty (Sirdeshmukh et al., 2002; Ling and Ding, 2006; Han et al., 2021). This was similar to findings in the field of education, where higher parental satisfaction was associated with increased loyalty to school (Leventhal et al., 2006; Chen, 2013; Budiyanto et al., 2021; Río-Rama et al., 2021). Thus, we reveal this positive relationship in the context of preschool education, which echoes previous research findings. The reason for this is that education has always been seen as an important issue for parents. Parents attach great importance to the quality of teaching and educational services provide by the school. This suggests that the more satisfied kindergarten parents are with the overall performance of their kindergarten, the closer it is to their expectations and the higher the level of parental loyalty.

According to H2, this study found that there was a partial mediation effect between parental satisfaction and parental loyalty in terms of the trust of kindergarten parents. This result was consistent with previous studies that satisfaction increases loyalty through the effect of parental trust (Oliva et al., 1992; Anderson and Sullivan, 1993; Song et al., 2019). The reason for this is that when kindergarten parents are satisfied with the kindergarten, they have more trust in the kindergarten, believe that the kindergarten will give priority to the children, and have confidence in the teaching environment provide by the kindergarten, and have a higher level of loyalty to the kindergarten.

According to H3, this study confirmed that there was a partial mediation effect between parental satisfaction and parental loyalty in relationship commitment of kindergarten parents. This finding was similar to previous research, where commitment played an important mediating role between satisfaction and loyalty (Skallerud, 2011; Jamshidi and Rousta, 2021). The reason for this is that when parents become satisfied with a kindergarten, a relationship commitment is created between the parents and the kindergarten. As relationship commitment serves as a symbol of the willingness of both parties to maintain a long-term, stable relationship, the more parents will be willing to devote extra time and effort to the kindergarten and take pride in their child’s attendance there, which in turn will strongly enhance parental loyalty.

According to H4, this study also found that kindergarten parental trust and parental relationship commitment played significant chain mediating roles in the relationship between parental satisfaction and parental loyalty, this was consistent with previous research findings according to that the chain meditation effect played a role in customers’ relationship and customers’ commitment (Sanchez-Franco et al., 2009). In addition, similar to empirical findings, trust and relationship commitment have been shown to be key mediating factors (Friman et al., 2002; Sue and Campbell, 2012; Wang and Lin, 2021). In other words, the KMV model can be applied to kindergarten parents, and trust and relationship commitment play a key mediating role between parental satisfaction and parental loyalty. Trust also positively influences relationship commitment. The reason for this is that the more satisfied parents are with the quality of teaching and educational services provided by a kindergarten, the more their trust and relationship commitment to the kindergarten will increase. By effectively acting as mediators through parental trust and parental relationship commitment, kindergartens will be able to secure the loyalty of parents, who will then be willing to keep their children in the kindergarten and recommend it to others.

### Theoretical Contributions

The results of this study provide a certain degree of theoretical contributions to the literature on preschool education. First, parental satisfaction had a significant and positive impact on parental loyalty in kindergartens. Secondly, both parental trust and parental relationship commitment played partial mediating roles in the relationship between parental satisfaction and parental loyalty. Thirdly, parental trust and parental relationship commitment played significant chain mediating roles in the relationship between parental satisfaction and parental loyalty in kindergartens. Previous research has established that satisfaction could influence loyalty (Sirdeshmukh et al., 2002; Ling and Ding, 2006; Han et al., 2021). There was also general support in the education literature for empirical findings on student or parental satisfaction and loyalty to schools (Leventhal et al., 2006; Chen, 2013; Budiyanto et al., 2021; Río-Rama et al., 2021). However, relatively little research has been conducted on the mechanisms by which student or parental satisfaction with the school influences loyalty referrals. In addition, a few empirical studies have been conducted in the past using Morgan and Hunt’s (1994) KMV model as a theoretical framework to investigate the mediating role of trust and relationship commitment in the relationship between customer satisfaction and loyalty in Internet services, and have been validated (Sanchez-Franco et al., 2009). However, up to now, empirical research on the mediating mechanisms through which parental satisfaction affects parental loyalty in kindergartens, using kindergarten parents as the main subject of study, has yet to be further explored. Therefore, the contribution of this study is that this study found that when kindergarten parents perceive higher levels of satisfaction, they exhibit higher levels of parental loyalty. Furthermore, parental trust and parental relationship commitment can function as useful mediating variables between parental satisfaction and parental loyalty. The results of this study enrich the relationship between kindergarten parental satisfaction and parental loyalty in the context of preschool education, which also promote the value of the KMV model in the application of preschool education.

### Practical Contributions

The findings of this study also provide some useful practical suggestions. First of all, kindergarten parental satisfaction had a significant and positive impact on parental loyalty. In practical terms, there are a number of ways in which kindergarten operators can approach this: Regular training and satisfaction monitoring of teachers so that the teaching and services provided by kindergartens meet parents’ expectations and parents feel that the cost is worth it. The process of interaction between teachers and parents should be pleasant so that parents are happy to choose the kindergarten. Kindergartens operators should conduct regular kindergarten satisfaction surveys with parents to identify possible causes of parental dissatisfaction and try to improve them.

Secondly, parental trust in kindergarten had a partial mediation effect between parental satisfaction and parental loyalty. Therefore, it is important to build up the trust of parents. For example, kindergarten teachers should communicate with parents in a timely manner about their children’s performance in kindergartens, so that parents can be confident that the kindergartens operators will be able to handle any unexpected incidents that occur. Kindergarten operators should enhance the teaching quality of teachers and provide pre-service and in-service training to teachers in kindergartens. Kindergartens operators should be safe and secure in terms of play facilities and regularly review the safety performance of their toy facilities, venues and large activity equipment. In terms of food and drink, kindergartens operators should display the licenses of their kitchen staff, strictly control the procurement and processing of food, take samples of every meal, and conduct regular cleaning checks on the kitchen to create an environment where parents can trust.

Thirdly, the results of this study also indicate that there was a partial mediation effect between parental satisfaction and parental loyalty in terms of the relationship commitment of kindergarten parents. Kindergarten operators should therefore focus on the following areas: kindergarten operators should organize more lively and enriching parent–child activities and encourage parents to participate. Kindergarten teachers need to communicate more with parents and become friends with them to maintain a long-term partnership; kindergarten operators should build a good image and share their teaching characteristics so that parents can feel honored to have their children in their kindergartens.

Finally, the findings of this study reveal that kindergarten parental trust and parental relationship commitment played significant chain mediating roles in the relationship between parental satisfaction and parental loyalty. Kindergarten operators therefore need to do a better job of promoting parental trust and parental relationship commitment if they are to ensure high levels of parental satisfaction and, in turn, high levels of parental loyalty. Parental trust can in turn positively influence parental relationship commitment. A good parental relationship commitment will help parents to be willing to keep their children or other children in the kindergarten, and to recommend the kindergarten to others. The chain mediation model developed in this study has a certain degree of practical contributions.

### Limits and Future Directions

However, there are still several limitations in this study. Firstly, this study uses convenience sampling and future studies can improve the sampling method to enhance the statistical power. Secondly, this study only discusses parental trust in kindergarten and parental relationship commitment as mediating variables between parental satisfaction and parental loyalty. Whether there are additional mediating variables in the influence process or whether the mediating variables are moderated by other variables remain to be further explored in future studies. Finally, this study is a cross-sectional study. The results of the study only confirm the relationship between the variables at the time. It is suggested that future studies could be conducted longitudinally to further understand the dynamic process of changing relationships between variables.

## Data Availability Statement

The raw data supporting the conclusions of this article will be made available by the authors, without undue reservation.

## Ethics Statement

The studies involving human participants were reviewed and approved by Dhurakij Pundit University. The patients/participants provided their written informed consent to participate in this study. Written informed consent was obtained from the individual(s) for the publication of any potentially identifiable images or data included in this article.

## Author Contributions

J-HH conceived the research idea and structured and drafted the manuscript. L-GC and Y-XM analyzed the data. S-YG participated in the revision of the manuscript. All authors read and approved the final manuscript and participated in the critical appraisal as well as revision of the manuscript.

## Conflict of Interest

The authors declare that the research was conducted in the absence of any commercial or financial relationships that could be construed as a potential conflict of interest.

## Publisher’s Note

All claims expressed in this article are solely those of the authors and do not necessarily represent those of their affiliated organizations, or those of the publisher, the editors and the reviewers. Any product that may be evaluated in this article, or claim that may be made by its manufacturer, is not guaranteed or endorsed by the publisher.
